# Triglyceride-glucose index trajectory and arterial stiffness: results from Hanzhong Adolescent Hypertension Cohort Study

**DOI:** 10.1186/s12933-022-01453-4

**Published:** 2022-02-25

**Authors:** Yu Yan, Dan Wang, Yue Sun, Qiong Ma, Keke Wang, Yueyuan Liao, Chen Chen, Hao Jia, Chao Chu, Wenling Zheng, Jiawen Hu, Yue Yuan, Yang Wang, Yuliang Wu, Jianjun Mu

**Affiliations:** 1 Department of Cardiology, First Affiliated Hospital of Medical School, Key Laboratory of Molecular Cardiology of Shaanxi Province, Ministry of Education, Xi’an Jiaotong University, Key Laboratory of Environment and Genes Related to Diseases (Xi’an Jiaotong University), NO.277, Yanta West Road, Xi’an, 710061 Shaanxi People’s Republic of China; 2 Department of Cardiovascular Surgery, First Affiliated Hospital of Medical School, Xi’an Jiaotong University, Xi’an, China; 3 Department of Cardiovascular Medicine, Jiangsu Province Hospital, Nanjing, China

**Keywords:** Triglyceride-glucose index, Insulin resistance, Arterial stiffness, Cohort study

## Abstract

**Background:**

The triglyceride-glucose index (TyG index) has emerged as a reliable surrogate marker of insulin resistance associated with arterial stiffness. However, most studies were based on a cross-sectional design, and few studies have evaluated the longitudinal impact of the TyG index on arterial stiffness. This study aimed to investigate the associations of single time point measurement and the long-term trajectory of the TyG index with arterial stiffness in a Chinese cohort.

**Methods:**

Data are derived from the Hanzhong Adolescent Hypertension Cohort study. A total of 2480 individuals who participated in the 2017 survey was included in the cross-sectional analysis. A sample of 180 individuals from the sub-cohort with follow-up data in 2005, 2013, and 2017 was enrolled in the longitudinal analysis. The TyG index was calculated as ln (fasting triglyceride [mg/dL] × fasting glucose [mg/dL]/2), and arterial stiffness was determined using brachial-ankle pulse wave velocity (baPWV). The latent class growth mixture modeling method was used to identify the TyG index trajectories from 2005 to 2017.

**Results:**

In the cross-sectional analysis, the median age of the study population was 42.8 (39.8, 44.9) years, and 1351 (54.5%) were males. Each one-unit increment in TyG index was associated with a 37.1 cm/s increase (95% confidence interval [CI] 23.7–50.6 cm/s; *P* < 0.001) in baPWV, and similar results were observed when the TyG index was in the form of quartiles. In the longitudinal analysis, we identified three distinct TyG index trajectories and found that the highest TyG index trajectory carried the greatest odds of increased arterial stiffness, with a fully adjusted odds ratio (OR) of 2.76 (95% CI 1.40, 7.54).

**Conclusions:**

Elevated levels of baseline TyG index and higher long-term trajectory of TyG index were independently associated with increased arterial stiffness. Monitoring immediate levels and longitudinal trends of the TyG index may help with the prevention of arterial stiffness in the long run.

**Supplementary Information:**

The online version contains supplementary material available at 10.1186/s12933-022-01453-4.

## Background

Arterial stiffness is considered one of the earliest detectable measures of vascular damage, strongly predicting cardiovascular morbidity and mortality [1–3]. Previously recognized predisposing factors of arterial stiffness include aging, elevated blood pressure, obesity, diabetes, and metabolic syndrome [4–6]. In particular, insulin resistance, a hallmark of obesity and metabolic syndrome, leads to oxidative responses, low-grade inflammation, and endothelial dysfunction, contributing to arterial stiffness progression [7–9]. As a surrogate marker of insulin resistance, the triglyceride-glucose (TyG) index, which is calculated as ln [fasting triglycerides (TG, mg/dL) × fasting blood glucose (FBG, mg/dL)/2], has been reported to be associated with cardiovascular risk [10–12]. The relationship between the TyG index and arterial stiffness, which is measured using brachial-ankle pulse wave velocity (baPWV), has also been reported. Emerging clinical studies have shown that an elevated TyG index is associated with higher values of baPWV and increased arterial stiffness [13–17]. However, most prior studies are cross-sectional design, and data are still limited regarding the dynamic change of the TyG index over time. Likewise, the associations between long-term TyG index trends and arterial stiffness are largely unknown.

In this study, we aim to investigate the associations between TyG index and arterial stiffness measured using baPWV in the Hanzhong Adolescent Hypertension Cohort Study. In addition, we intended to identify long-term TyG index trajectories and characterize their association with arterial stiffness.

## Methods

### Study population

**Fig. 1:**
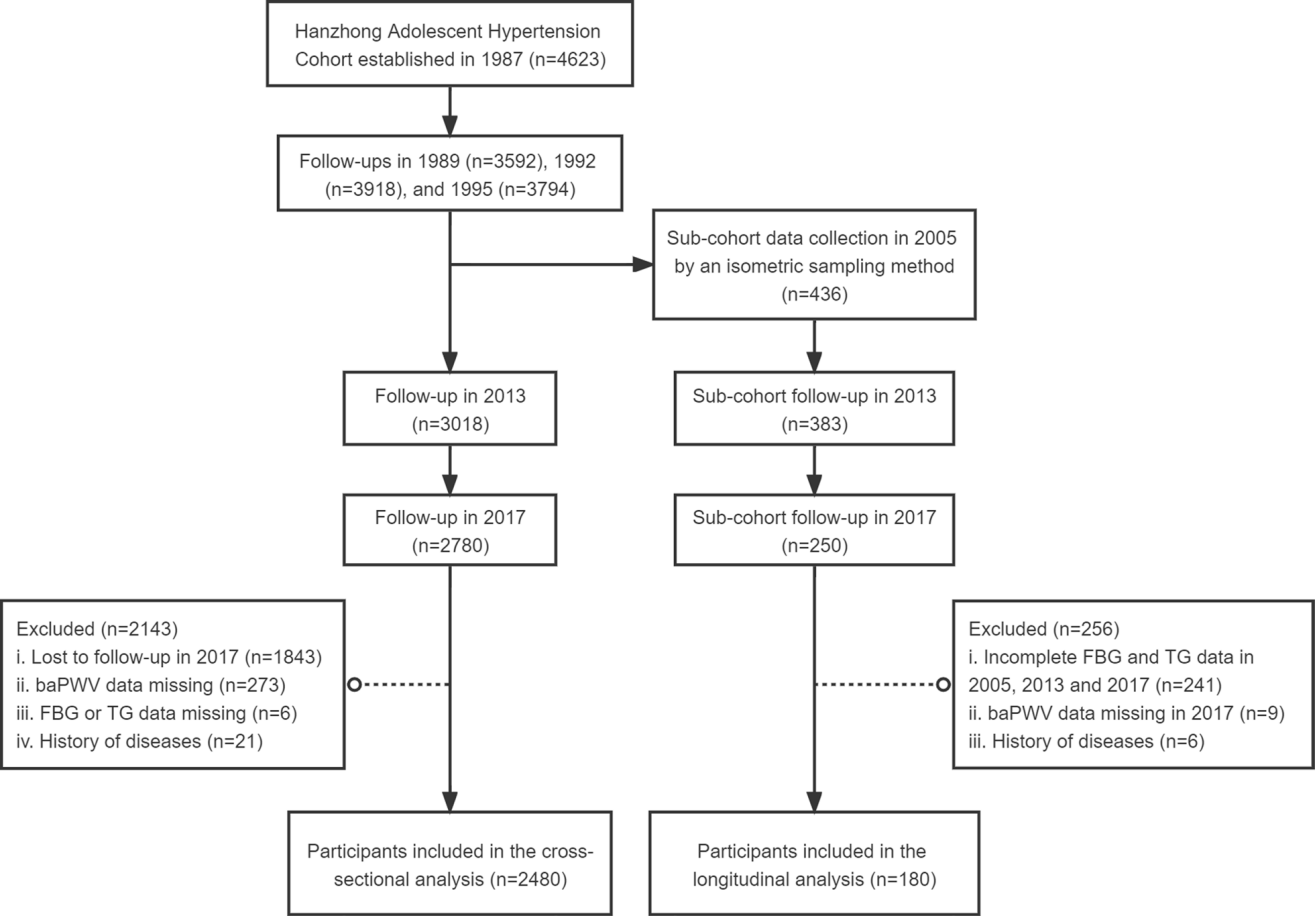
Flowchart for inclusion/exclusion of study participants

The participants were derived from the Hanzhong Adolescent Hypertension Cohort, an ongoing prospective cohort established in 1987 focusing on the natural development of cardiovascular risk factors, which has been described in detail elsewhere [18]. Briefly, in March and April 1987, 4623 schoolchildren aged 6 to 15 years were enrolled from 26 rural sites in Hanzhong, China, and a series of follow-ups were conducted in the following 30 years. In 2005, a subcohort of 436 participants was randomly selected using an isometric sampling method (*K* = 10) [19–21]. In the present study: (1) individuals who participated in the latest examination in 2017 and received the baPWV and laboratory test (N = 2480) were enrolled in the cross-sectional analysis; (2) individuals in the sub-cohort received baPWV test in 2017 with complete FBG and TG data in 2005, 2013 and 2017 (N = 180) were included in the longitudinal analysis. During the selection, individuals who had a history of myocardial infarction, heart failure, stroke, renal failure, or peripheral artery disease were excluded from the analysis (Fig. 1).

This study was approved by the Ethics Committee of the First Affiliated Hospital of Xi’an Jiaotong University (XJTU1AF2015LSL-047) and was clinically registered (NCT02734472, 12/04/2016). Written informed consent was obtained from all participants.

### Characteristics and definition

Demographic characteristics, lifestyle information, use of medicines, and medical history were collected using a standard questionnaire; height, weight, waist circumference, and hip circumference were measured using standardized protocols by trained staff at each visit [20]. Systolic and diastolic blood pressures (SBP, DBP) were measured in the seated position after resting for at least 5 min by experienced technicians using mercury sphygmomanometers or electric devices (Omron M6; Omron, Kyoto, Japan). The average of the three blood pressures readings was used for analysis. Body mass index (BMI) was calculated as the weight (in kilograms) divided by the square of the height in meters (kg/m^2^). Hypertension was defined as systolic and/ or diastolic blood pressures ≥ 140/90 mmHg or the current use of antihypertensive medications. Diabetes was defined as FBG ≥ 7.0 mmol/L in the cohort exam or self-reporting a history of diabetes diagnosed by physicians. Dyslipidemia was defined as the presence of any one of the following situations: hypertriglyceridemia (TG ≥ 2.26 mmol/L), hypercholesterolemia (TC ≥ 6.22 mmol/L), high levels of low-density lipoprotein cholesterol (LDL-C ≥ 4.14 mmol/L), or low levels of high-density lipoprotein cholesterol (HDL-C < 1.04 mmol/L). Cigarette smoking and alcohol drinking were defined as continuous or cumulative smoking for at least 6 months and consuming alcohol every day and for 6 months. Regular exercise was defined as taking part in physical exercise more than once a week [22].

### Biochemical parameters

Fasting blood samples were obtained in the morning, and a range of biochemical parameters was analyzed using an automatic biochemical analyzer (Hitachi 7600; Hitachi, Tokyo, Japan). The biochemical parameters tested included FBG, TC, TG, LDL-C, HDL-C, serum uric acid, serum creatinine, and high-sensitivity C-reactive protein (hs-CRP). TyG index was calculated as ln [fasting triglyceride (mg/dL) × fasting glucose (mg/dL)/2] [17].

### BaPWV measurement

Measurement of baPWV was performed using a volume-plethysmographic apparatus (BP-203RPEII; Nihon Colin, Tokyo, Japan), as detailed elsewhere [18, 23]. Briefly, measurements were performed for each participant in a supine position after a 5 min rest, with cuffs wrapped around both sides of the upper arm and ankle, ECG electrodes placed on both wrists, and a heart sound sensor placed on the fourth intercostal space left sternal border. The path distance from the suprasternal notch to the brachium (Lb) or to the ankle on the same side (La), and the time interval between the brachial and ankle waveforms (ΔT) were obtained automatically, and baPWV was calculated as baPWV (cm/s) = (La−Lb)/ ΔT. In this study, the mean value of the right and left baPWV was used for analysis, and a value of baPWV ≥ 1400 cm/s was considered as high-risk baPWV according to the Framingham Risk Score [23, 24].

### Statistical methods

Participants’ characteristics were described according to the quartile groups of the TyG index in the cross-sectional analysis, and according to the TyG index trajectories in the longitudinal analysis. Data were presented as the mean ± standard deviation, the medians with interquartile ranges, and frequencies or percentages where applicable. Linear trends in characteristics among groups were investigated using one-way ANOVA for linear trends and the Jonckheere-Terpstra test for continuous variables with normal and skewed distributions, and using the chi-square test for linear trend (Cochran–Armitage trend test) for categorical variables. Differences in baPWV values across quartiles were tested using Kruskal–Wallis test and post hoc test. The dose–response relationship between the TyG index and baPWV in the cross-sectional analysis was explored using restricted cubic regression with three knots located at percentiles (10th, 50th, and 90th) of the TyG index. The median of the TyG index was used as the reference point [17]. Multivariate linear regression models were used to estimate the associations of the TyG index with baPWV values in the cross-sectional analysis, with confounding variables selected based on previous literature and clinical knowledge [13, 17]. Several models were fitted on unadjusted analyses to determine the association between arterial stiffness and the TyG index according to prior published literature [13]. The long-term TyG index trajectories were identified using the latent class growth mixture modeling method [20]. Participants with similar patterns of change in the TyG index from 2005 to 2017 were identified and assigned to corresponding groups. Models were fitted using the LCMM package (version 1.8.1) in R (version 3.6.1, Vienna, Austria). The optimal shape of trajectories (intercept, linear, or quadratic) and the number of groups (starting with 2-group) were determined by: (1) higher Bayesian information criterion, (2) at least 5% of the total membership in each trajectory, (3) higher mean posterior probabilities (> 0.7) [25, 26]. Finally, 3 distinct trajectories turned out to be the best-fitting model. The associations of the TyG index with baPWV values in the cross-sectional and the longitudinal analysis were estimated using multivariate linear regression models and the logistic regression models, respectively. Logistic regression models were utilized to investigate the associations between TyG index trajectory and arterial stiffness, with candidate variables selected using stepwise selection methods. To assess the robustness of the associations between TyG index trajectories and arterial stiffness, we also conducted a sensitivity analysis by excluding participants who were on any antihypertensive, antidiabetic, or lipid-lowering medications. All analyses were conducted in R and SPSS software (version 22.0, SPSS Inc., Chicago, IL). A two-sided P value < 0.05 was considered statistically significant.

## Results

### Characteristics of the study participants in the cross-sectional analysis

Among the 2480 eligible participants, the median age was 42.8 (39.8, 44.9) years, and 1351 (54.5%) were males. The median TyG index was 8.5 (8.1, 8.9), and the median baPWV was 1215.5 (1091.5, 1373.0) cm/s. Participants were stratified into four groups on the basis of the TyG index levels (Table 1). Compared to those in the lowest TyG index quartile, participants in the higher quartiles were more often to be men, smokers, drinkers, and had a higher prevalence of hypertension, diabetes, and dyslipidemia. In addition, BMI, heart rate, SBP, DBP, waist/hip ratio, FBG, TC, TG, LDL-C, serum uric acid, serum creatinine, and hs-CRP level were all positively correlated with increasing TyG index quartiles, while HDL-C was negatively correlated (all *P* for trend < 0.05).

**Table 1:** Characteristics of participants according to quartiles of TyG index in 2017 (n = 2480)

Characteristics	Quartiles of TyG index				*P* for trend
	Q1 (5.71–8.14)	Q2 (8.14–8.49)	Q3 (8.49–8.90)	Q4 (8.90–12.31)	
N	620	620	620	620	
Age, years^a^	42.8 (39.7, 44.9)	42.8 (39.4, 44.9)	43.1 (39.9, 45)	42.6 (40, 44.8)	0.988
Male, n (%)^b^	236 (38.1%)	299 (48.2%)	370 (59.7%)	446 (71.9%)	< 0.001
Regular exercise, n (%)^b^	363 (58.5%)	373 (60.2%)	365 (58.9%)	363 (58.5%)	0.866
Smoking, n (%)^b^	179 (28.9%)	232 (37.4%)	284 (45.8%)	346 (55.8%)	< 0.001
Drinking, n (%)^b^	117 (18.9%)	155 (25.0%)	209 (33.7%)	231 (37.3%)	< 0.001
BMI, kg/m^2a^	22.1 (20.6, 23.9)	23.1 (21.5, 24.9)	24.6 (22.5, 26.6)	25.5 (23.6, 27.8)	< 0.001
Waist hip ratio^a^	0.9 (0.8, 0.9)	0.9 (0.9, 0.9)	0.9 (0.9, 1)	1.0 (0.9, 1)	< 0.001
Heart rate, bpm^a^	72 (65, 78)	72 (67, 79)	73 (67, 80)	75 (68, 82)	< 0.001
SBP, mmHg^a^	116.3 (108.7, 126.0)	118.3 (110, 128)	123.5 (114.6, 132.7)	126.3 (117.3, 137)	< 0.001
DBP, mmHg^a^	72 (66.7, 79)	74 (67, 81.3)	77.5 (70.3, 85)	80.7 (74, 88.3)	< 0.001
FBG, mmol/L^a^	4.4 (4.1, 4.7)	4.5 (4.2, 4.8)	4.6 (4.3, 4.9)	4.8 (4.5, 5.4)	< 0.001
TC, mmol/L^a^	4.2 (3.8, 4.7)	4.4 (4, 4.9)	4.6 (4.2, 5)	4.8 (4.4, 5.3)	< 0.001
TG, mmol/L^a^	0.8 (0.7, 0.9)	1.1 (1, 1.3)	1.6 (1.4, 1.8)	2.5 (2.2, 3.3)	< 0.001
LDL-C, mmol/L^a^	2.3 (2, 2.6)	2.5 (2.2, 2.9)	2.6 (2.3, 3)	2.6 (2.1, 3)	< 0.001
HDL-C, mmol/L^a^	1.3 (1.2, 1.5)	1.2 (1.1, 1.4)	1.1 (1, 1.2)	1.0 (0.9, 1.1)	< 0.001
Serum uric acid, μmol/L^a^	238.6 (200.5, 287.6)	264.5 (215.6, 312.4)	293.9 (244.6, 346.2)	323.1 (268.3, 374.8)	< 0.001
Serum creatinine, μmol/L^a^	71.3 (63.2, 81)	74.3 (65.5, 85)	77.4 (68.2, 86.8)	80.6 (71.6, 89.5)	< 0.001
TyG index^a^	7.9 (7.8, 8)	8.3 (8.2, 8.4)	8.7 (8.6, 8.8)	9.2 (9.1, 9.5)	< 0.001
hs-CRP, μmol/L^a^	0.2 (0.2, 0.6)	0.2 (0.2, 0.6)	0.3 (0.2, 0.8)	0.3 (0.2, 0.8)	< 0.001
baPWV, cm/s^a^	1160 (1047.3, 1294)	1174 (1071.5, 1301)	1241 (1109.8, 1404.3)	1299.8 (1169.5, 1468)	< 0.001
Hypertension, n (%)^b^	52 (8.4%)	68 (11.0%)	111 (17.9%)	163 (26.3%)	< 0.001
Diabetes, n (%)^b^	0	2 (0.3%)	4 (0.6%)	59 (9.5%)	< 0.001
Dyslipidemia, n (%)^b^	58 (9.4%)	139 (22.4%)	228 (36.8%)	527 (85.0%)	< 0.001

*BMI* body mass index, *SBP* systolic blood pressure, *DBP* diastolic blood pressure, *FBG* fasting blood glucose, *TC* total cholesterol, *TG* triglyceride, *LDL-C* low-density lipoprotein cholesterol, *HDL-C* high-density lipoprotein cholesterol, *TyG index* triglyceride–glucose index, *hs-CRP* high-sensitivity C-reactive protein, *baPWV* brachial-ankle pulse wave velocity

^a^Data are given as median (interquartile range), and *P* values for trend were calculated by the Jonckheere-Terpstra test

^b^Data are expressed as number (percentage), and *P* values for trend were calculated using Cochran–Armitage trend test

### Association of the TyG index with baPWV

**Fig. 2:**
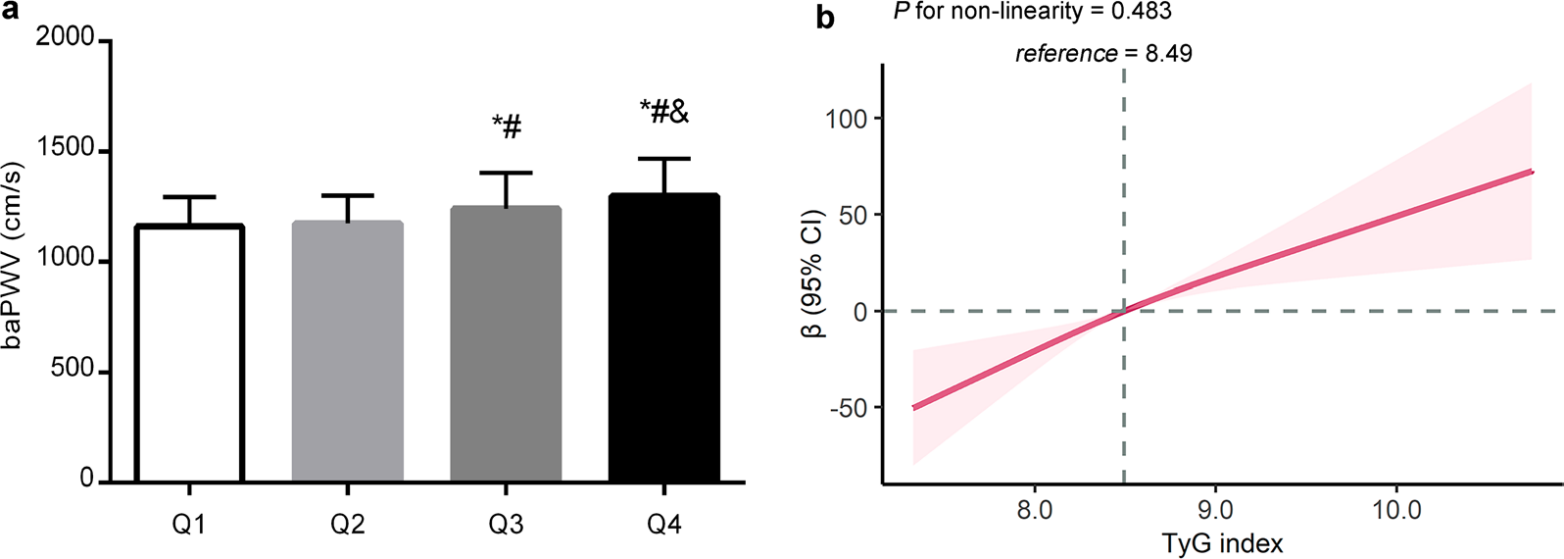
Comparison of the baPWV levels among the TyG index quartiles (**a**) and the associations of the TyG index with baPWV (**b**). **a** Differences in baPWV values across quartiles were tested using Kruskal–Wallis test and post hoc test. *P < 0.05 versus the first quartile; #*P* < 0.05 versus the second quartile; &*P* < 0.05 versus the third quartile. **b** Data were fitted with a linear regression model using restricted cubic splines with 3 knots at 10, 50, and 90th percentiles of TyG index. The model was adjusted for age, sex, smoking, drinking, regular exercise, SBP, BMI, hs-CRP, and diabetes. The solid line represented the estimations, and the shaded area represented 95% confidence interval

The mean baPWV values increased with increasing quartiles of the TyG index (*P* < 0.05, Fig. 2a). Overall, baPWV was positively associated with the TyG index (Fig. 2b). For linear regression models measuring TyG index as a continuous variable, each one-unit increment in TyG index was associated with a 37.1 cm/s increase (95% CI, 23.7–50.6 cm/s; *P* < 0.05) in baPWV, after adjustment for age, sex, smoking, alcohol drinking, regular activity, BMI, hs-CRP, SBP, and diabetes status (Table 2). Likewise, the categorical analysis revealed that compared with those in the lowest quartile of TyG index, the third and highest quartile of TyG index were significantly associated with a 38.4 cm/s (95% CI 17.2–59.7 cm/s; *P* < 0.05) and 67.3 (95% CI 42.8–91.8 cm/s; *P* < 0.05) increase in baPWV after adjusting for all covariates, respectively.

**Table 2:** Association of TyG index with baPWV in 2017 (n = 2480)

TyG index	Unadjusted		Model 1		Model 2	
	β (95% CI)	P value	β (95% CI)	P value	β (95% CI)	P value
Per 1 unit increase	105.4 (90.0, 120.8)	< 0.001	82.8 (67.6, 97.9)	< 0.001	37.1 (23.7, 50.6)	< 0.001
Q1 (5.71–8.14)	Reference		Reference		Reference	Reference
Q2 (8.14–8.49)	21.8 (− 1.4, 45.0)	0.065	13.8 (− 8.4, 36.0)	0.224	14.2 (-4.0, 32.3)	0.126
Q3 (8.49–8.90)	92.3 (66.9, 117.8)	< 0.001	68.1 (43.1, 93.1)	< 0.001	38.4 (17.2, 59.7)	< 0.001
Q4 (8.90–12.31)	159.9 (133.4, 186.5)	< 0.001	131.1 (104.0, 158.2)	< 0.001	67.3 (42.8, 91.8)	< 0.001

Model 1, adjusted for sex and age

Model 2, further adjusted for smoking, alcohol drinking, regular exercise, BMI, SBP, hs-CRP, and diabetes in 2017

### Baseline characteristics of the study participants in the longitudinal study

**Fig. 3:**
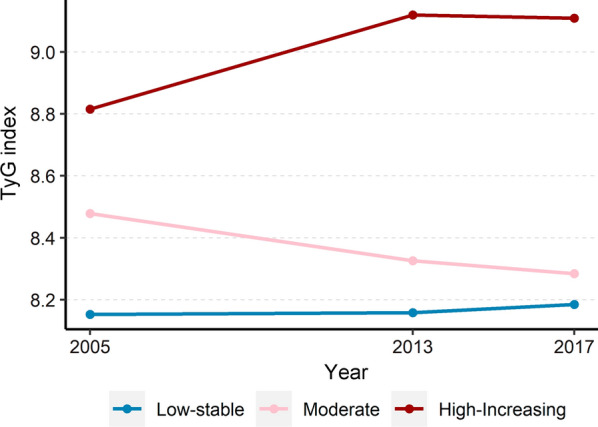
TyG index trajectory over a 12-year follow-up

Among 180 participants included in the longitudinal analysis, the median age was 28.7 (27.1–32.2) years at baseline, and 108 (60.0%) were men. Over the 12-year observation period, three distinct TyG index trajectories were identified: low-stable (n = 63, 35.0%) moderate (n = 46, 25.6%), and high-increasing (n = 71, 39.4%; Fig. 3) TyG index trajectory groups. At baseline, the TyG index levels for each dynamic trajectory group were 8.2 ± 0.4, 8.5 ± 0.2, and 8.8 ± 0.5, respectively (Table 3). Compared with the low-stable group, participants in the moderate and high-increasing groups tended to be males, had higher BMI and waist/ hip ratio, and had higher blood pressures, FBG, TC, TG, LDL-C, and TyG index (all *P* for trend < 0.05). No significant difference was observed in age, HDL-C level, hs-CRP, and prevalence of hypertension and dyslipidemia among the three groups. No participant was found with diabetes at baseline. A comparison of characteristics of study participants in survey 2017 among the three trajectory groups is presented in Additional file 1: Table S1. Significant differences were observed in lifestyles, anthropometric parameters, medical history, and laboratory indices. Notably, participants in the high-increasing groups were more likely to have higher TyG index levels and baPWV values relative to their counterparts in the low-stable group (*P* for trend < 0.05). The median changes in TyG index level over the 12 years were 0.06 (− 0.10 to 0.25) in the low-stable group, -0.15 (− 0.28 to 0.04) in the moderate group, and 0.28 (− 0.14 to 0.67) in the high-increasing trajectory group.

**Table 3:** Baseline characteristics of participants in 2005 according to TyG index trajectory (n = 180)

Characteristics	Trajectory of TyG index			*P* for trend
	Low-stable	Moderate	High-increasing	
N	63	46	71	
Age, years^a^	28.8 (27, 32.6)	29.4 (27.4, 32.5)	28.2 (26.5, 30.6)	0.067
Male, n (%)^b^	28 (44.4%)	24 (51.1%)	56 (80.0%)	< 0.001
BMI, kg/m^2a^	21.6 (19.9, 24.4)	21.9 (20.1, 23.5)	23.9 (21.6, 25.9)	< 0.001
Waist hip ratio^a^	0.8 (0.8, 0.9)	0.8 (0.8, 0.9)	0.9 (0.8, 0.9)	< 0.001
Heart rate, bpm^a^	70 (66, 76)	74 (68, 79)	76 (68, 80)	0.014
SBP, mmHg^c^	119.1 ± 13.6	121.2 ± 13.7	126.3 ± 14.7	0.004
DBP, mmHg^c^	76.5 ± 10.5	76.1 ± 10.3	82.4 ± 9.8	0.001
FBG, mmol/L^a^	4.6 (4.3, 5.0)	4.6 (4.3, 5.2)	4.9 (4.4, 5.5)	0.007
TC, mmol/L^c^	4.2 ± 0.7	4.3 ± 0.6	4.6 ± 0.7	< 0.001
TG, mmol/L^a^	0.9 (0.7, 1.1)	1.3 (1.1, 1.4)	1.8 (1.3, 2.2)	< 0.001
LDL-C, mmol/L^a^	2.5 (2.2, 2.8)	2.6 (2.3, 2.8)	2.6 (2.5, 3.1)	0.005
HDL-C, mmol/L^a^	1.1 (0.9, 1.2)	1.1 (1, 1.2)	1.1 (1, 1.3)	0.065
TyG index^c^	8.2 ± 0.4	8.5 ± 0.2	8.8 ± 0.5	< 0.001
hs-CRP, μmol/L^a^	0.3 (0.2, 0.6)	0.3 (0.2, 0.7)	0.4 (0.2, 1.7)	0.121
Hypertension, n (%)^b^	9 (14.3%)	7 (14.9%)	15 (21.4%)	0.270
Dyslipidemia, n (%)^b^	30 (47.6%)	17 (37.0%)	32 (45.1%)	0.793

^a^Data are given as median (interquartile range), and *P* values for trend were calculated by the Jonckheere-Terpstra test

^b^Data are expressed as number (percentage), and *P* values for trend were calculated using Cochran–Armitage trend test

^c^Data are shown as mean ± standard deviation, and P values for trend were calculated by one-way analysis of variance for linear trend

### Association of TyG index trends with arterial stiffness

Among the 180 participants, 39 (21.7%) had increased baPWV in 2017. The incident rate presented an increasing trend, which was 7 (11.1%), 11 (23.4%), and 21 (30.0%) in the low-stable, moderate, and high-increasing trajectory groups, respectively. The association between the TyG index trajectories and increased arterial stiffness is presented in Table 4. Taking the low-stable group as a reference, participants in the high-increasing group had over 3.3 times the odds of arterial stiffness (OR = 3.32 (1.24–8.90); *P* = 0.017), based on age-sex-adjusted multivariable logistic regression analysis. This relationship remained statistically significant after further adjustment for regular exercise and hypertension. Participants in the moderate group showed a comparable probability of arterial stiffness to those in the low-stable group (OR = 2.39 (0.85–6.77); *P* = 0.10), and further adjustments produced similar results.

**Table 4:** Association of TyG index trajectories with high-risk baPWV in 12-year follow-ups

TyG index trajectories	High-risk baPWV (%)	Unadjusted		Model 1		Model 2	
		OR (95% CI)	*P* value	OR (95% CI)	*P* value	OR (95% CI)	*P* value
Low-stable	7 (11.1%)	Reference		Reference		Reference	
Moderate	11 (23.4%)	2.44 (0.87, 6.89)	0.091	2.39 (0.85, 6.77)	0.100	2.51 (0.85, 7.39)	0.095
High-increasing	21 (30.0%)	3.43 (1.34, 8.75)	0.01	3.32 (1.24, 8.90)	0.017	2.76 (1.40, 7.54)	0.037

Model 1, logistic regression adjusted for sex and age

Model 2, further adjusted for regular exercise and hypertension

### Sensitivity analysis

Excluding participants who were receiving antihypertensive, antidiabetic, or lipid-lowering treatments (n = 22) did not lead to substantial changes to the results (Additional file 2: Table S2). Interestingly, excluding participants with active treatments strengthened the association between TyG index trajectories with arterial stiffness; relative to the low-stable group, the high-increasing group had the highest odds for arterial stiffness (OR = 3.57 (1.45, 9.63), *P* = 0.037), and the moderate group showed a borderline significant association with arterial stiffness (OR = 3.54 (1.00, 9.59), *P* = 0.051) after adjustment for various confounders.

## Discussion

In the present study, we observed a significant association between the TyG index and baPWV as a measurement of arterial stiffness in the general population. In cross-sectional settings, the TyG index positively correlated with baPWV in both continuous variables and quartiles form. Furthermore, during a 12-year observation period, 3 heterogeneous patterns of TyG index trajectory were identified, with the high-increasing trajectory carrying the greatest odds of increased arterial stiffness. These findings suggest that high levels of insulin resistance, as well as long-term exposure to high insulin resistance, may play a role in the pathogenesis of arterial stiffness.

TyG index is a surrogate marker of insulin resistance deemed to provide better predictive performance in cardiovascular disease. Mounting evidence from cohort studies has confirmed the elevated TyG index to be a reliable predictor of adverse cardiovascular events [10–12, 27–30]. As of late, the association between the TyG index and arterial stiffness has gained increasing attention. Lee et al. demonstrated that elevated TyG index independently associated with increased arterial stiffness as measured by baPWV in the Korean general population [13]. Likewise, Guo et al. reported the TyG index to be an independent risk factor for high baPWV among Chinese healthy individuals [15]. Emerging evidence also suggested that the TyG index was positively and independently associated with baPWV in older adults, diabetics, and hypertensive patients [14, 16, 31, 32]. Additionally, Wu et al. reported a longitudinal relationship between the baseline TyG index and arterial stiffness progression in the Kailuan cohort, indicating that a higher TyG index predicted a higher risk and faster progression of arterial stiffness [17]. Despite the abundance of evidence for a cross-sectional association between the TyG index and baPWV, our investigation has a few differences from previous studies. One of the most notable differences is the population composition. Our study consisted of participants from a population-based cohort, with diverse socio-economic characteristics [13, 15, 17]. Another difference is that the individuals included in this study were relatively young, with a mean age of 42 years. Middle adulthood (age 40 to 65 years) is the time where cardiovascular profile rapidly changes, and is also an important life stage for early detection and intervention of cardiovascular risk. With these points in mind, our study based on a general population suggests that the TyG index can also function as a useful early indicator of arterial stiffness and thus reflects cardiovascular risk in early middle age. Previous studies were mainly focused on the TyG index measured at a single time point, which may not capture long-term exposure. Considering the dynamic changes in TG, FBG, and therefore TyG index over time, the impact of longitudinal TyG index trends needs to be evaluated. In a recent study, Gao et al. observed 4 distinct trajectories of the TyG index over 20 years, each associated with a different risk of future peripheral artery disease, with the two-decade trajectory with an elevated TyG index presenting the greatest risk [33]. However, little is known about the longitudinal variation of the TyG index and its association with subsequent arterial stiffness. Consistent with previous studies, our findings confirmed a significant cross-sectional association of the TyG index with baPWV. Importantly, we expanded previous observations by identifying distinct TyG index trajectories from early to middle adulthood. To the best of our knowledge, this is the first report concerning the impact of long-term TyG index trajectory on the progression of arterial stiffness in the general population. We highlighted the fact that the TyG index could fluctuate over time, and a single time point measurement cannot fully reflect the heterogeneous patterns of trends. Long-term trajectories of the TyG index provide additional information about the cumulative burden of risk for arterial stiffness. Our findings further suggested that participants with consistently higher TyG index (mean TyG index > 8.8) from early to middle adulthood were more susceptible to increased arterial stiffness after adjustment for traditional risk factors. In clinical practice, such individuals require early and intensive screening and management of risk factors to prevent arterial stiffness and, consequently, cardiovascular events.

Although the underlying mechanisms accounting for the associations between the TyG index and arterial stiffness have not been fully elucidated, it appears to be related to insulin resistance. The insulin signaling pathway could be disturbed by insulin resistance, leading to oxidative stress and impaired endothelial function at the intima cells level, including endothelial cells, smooth muscle cells, and macrophages. Furthermore, insulin action could also be impaired by insulin resistance, promoting chronic low-grade inflammation and dysregulation of glucose homeostasis [34–36]. These processes contribute to the development of arterial stiffness. Our results also confirmed previous findings that participants with high TyG index quartiles showed higher hs-CRP levels, a sensitive marker of inflammation, suggesting inflammation may be involved in the interplay between insulin resistance and arterial stiffness [17]. In 2008, Simental-Mendía et al*.* firstly introduced the TyG index as a surrogate to identify insulin resistance in apparently healthy subjects [37]. Growing evidence suggests that the TyG index performs better for assessing insulin resistance and is better associated with CVD and arterial stiffness than the homeostasis model assessment for insulin resistance (HOMA-IR), which is a traditional and the most frequently used marker of insulin resistance [13, 31, 38–41]. The molecular mechanisms of the TyG index as an effective marker of insulin resistance have not been completely understood, but it may be related to impaired metabolic flexibility. Insulin resistance disrupts insulin-mediated suppression of glucose production, and perturbs fatty acid metabolism during accumulation of skeletal muscle triglyceride [42, 43]. Nevertheless, the underlying mechanisms still warrant further investigation at the pathway levels.

Our study also has several limitations that need to be acknowledged. First, the study population was restricted to northern Chinese individuals, limiting the generalizability of the findings. Second, the follow-ups were unequally spaced in time, resulting in a shortage of data from 2005 to 2013. Third, the sample of participants in the longitudinal analysis was relatively small, leading to the less precise estimation of the parameters, which was reflected as wide confidence intervals for ORs. And during the long-term follow-up, there is a loss of participants. However, baseline characteristics in 2005 were similar between the participants and non-participants, and the study cohort seems to be representative of the original study population (see Additional file 3: Table S3, Additional file 4: Table S4). Additionally, participants included in this study were relatively young, with the oldest being 45 years old at the latest follow-up, and the number of participants with high baPWV was rather small. Large-scale prospective population studies are required to further understand and confirm the conclusions. Despite these limitations, this is the first study to demonstrate the association between TyG index trajectories and arterial stiffness. The combination of cross-sectional and longitudinal analyses provides a comprehensive insight into this association.

## Conclusions

Participants with elevated levels of baseline TyG index and higher long-term trajectory of TyG index were more likely to have a higher risk of arterial stiffness. These findings emphasize the importance of long-term TyG index patterns in the evolution of arterial stiffness and may help with the early detection of high-risk individuals.

## Supplementary Information

**Additional file 1:** **Table S1.** Characteristics of participants in 2017 according to TyG index trajectory (n=180).

**Additional file 2:** **Table S2.** Association of TyG index trajectories with high-risk baPWV in 12-year follow-ups (sensitivity analysis).

**Additional file 3:** **Table S3.** Comparison of characteristics in 1987 between participants and non-participants in the cross-sectional study.

**Additional file 4:** **Table S4.** Comparison of characteristics in 2005 between participants and non-participants in the longitudinal study.

## Data Availability

The derived data generated in the current study are available from the corresponding author, upon reasonable request.
